# Shengjie Tongyu Granule Inhibits Vascular Remodeling in ApoE-Gene-Knockout Mice

**DOI:** 10.1155/2012/897875

**Published:** 2012-06-28

**Authors:** Min Chen, Wenli Cheng, Zaixiang Shi, Nishihara Tatsukazu, Tongliang Zhou, Hong Li, Jian Guo

**Affiliations:** ^1^Center for Cardiovascular Diseases, China-Japan Friendship Hospital, Ministry of Health, Beijing 100029, China; ^2^Graduate School, Beijing University of Traditional Chinese Medicine, Beijing 100029, China; ^3^Instutite of Clinical Studies, China-Japan Friendship Hospital, Ministry of Health, Beijing 100029, China

## Abstract

The aim of the present paper was to investigate the effect of Shengjie Tongyu granule on vascular remodeling in atherosclerotic mice and the relevant underlying mechanism. Sixty male ApoE-gene-knockout mice, fed a high-fat diet from 6 weeks of age, were randomized into a Shengjie Tongyu granule group (4.00 g/kg/d), a simvastatin group (9.01 mg/kg/d), and a control group (normal saline: 0.2 mL/d). At the ages of 30 and 40 weeks, we sacrificed the mice for various measurements. The results show that treatment with Shengjie Tongyu granule and simvastatin significantly decreased lumen and plaque areas in the aortic root at 30 and 40 weeks of age, decreased grade II elastic fiber lesions in the ascending aorta at 30 weeks of age, and decreased both grade II and III lesions at 40 weeks of age, compared to controls. The content of superoxide anions, and expression of MOMA-2, plasma ICAM-1, and NF**κ**B p50 in 30- and 40-week-old mice in the Shengjie Tongyu granule and simvastatin groups were also significantly reduced compared to the control group. In conclusion, Shengjie Tongyu granule has a clear inhibitory effect on vascular remodeling and on inflammatory pathways in ApoE-gene-knockout mice.

## 1. Introduction

Atherosclerosis is generally considered to be an inflammatory proliferative disease. Vascular remodeling, a dynamic process of conformational changes in the vasculature resulting from cell proliferation, necrosis and migration; and extracellular matrix synthesis and degradation, plays an important role in the development of atherosclerosis, inflammation and oxidation play an important role in vascular remodeling [1, 2]. In atherosclerotic vessels undergoing remodeling, increased numbers of inflammatory cells including macrophages and T lymphocytes, increased matrix metalloproteinase (MMP) expression, decreased vascular smooth muscle mass, decomposition of elastin and collagen, and an attenuated vascular wall, all closely related to changes in the redox state of the cells, are found [3]. Production of local reactive oxygen species (superoxide anions, nitric oxide, etc.) can increase low-density lipoprotein (LDL) oxidation, local inflammation, vascular adventitial fibroblast proliferation, and extracellular matrix synthesis. Local reactive oxygen species can also directly activate NF*κ*B and stimulate the expression of NF*κ*B-dependent genes, including genes of proinflammatory factors related to atherosclerosis, such as intercellular adhesion molecule 1 (ICAM-1). The increase in expression of proinflammatory genes then further increases the atherosclerotic process and vascular remodeling [3, 4]. 

 Because vascular remodeling plays an important role in atherosclerotic disease and patient prognosis, exploration of methods to improve the vascular remodeling process has been a subject of intensive research in recent years [5, 6]. Statins are one type of substance shown to have a beneficial effect on vascular remodeling. The mechanism of simvastatin in improving vascular remodeling has been ascribed to its multiple actions, especially its anti-inflammatory properties, rather than to its direct lipid-lowering effect [7].

Chinese herbal medicines are known to be effective in treating atherosclerosis [8], and their effects in improving vascular remodeling warrant further exploration. Shengjie Tongyu granule, on the basis of Sheng Xian Tang introduced by a famous physician in the Qing Dynasty, contains a number of bioactive components. Of these components, astragalus polysaccharide exerts an antiatherosclerotic effect through inhibiting TNF-alpha-induced activation of foam cells [9], and timosaponin affects calcium mobilization in vascular endothelial cells and smooth muscle cells and regulates vascular tension [10], and inhibits superoxide production and platelet aggregation after neutrophil activation [11–13]. Therefore, it is a reasonable hypothesis that Shengjie Tongyu granule might be efficacious for treating atherosclerotic diseases. Because the Shengjie Tongyu granule has antiatherosclerotic, anti-inflammatory, and antiplatelet aggregation effects, we speculated that the antiatherosclerotic effect of Shengjie Tongyu granule is related to its anti-inflammatory actions. 

The ApoE-gene-knockout mice mouse is a suitable animal model for study of atherosclerosis. ApoE is a constituent of plasma lipoproteins and serves as a ligand for cell-surface lipoprotein receptors such as the LDL-receptor (LDLr) that promote the uptake of atherogenic particles from the circulation. Homozygous deletion of the ApoE gene in mice results in a pronounced increase in the levels of plasma LDL and very low-density lipoprotein, due to the failure of LDLr- and lipoprotein receptor-related protein-mediated clearance of these lipoproteins. The most clearly observable phenotype of ApoE-gene-knockout mice is the spontaneous development of atherosclerotic lesions [14] which highly resemble human atherosclerosis [15] combined with the spontaneous development of arterial vascular remodeling [16].

In the current study, we used ApoE-gene-knockout mice as an animal model of atherosclerosis and simvastatin as a positive control to investigate the inhibitory effect of Shengjie Tongyu granule on vascular remodeling in atherosclerotic arteries and to see if the mechanism of its effect is related to a decrease in oxidation and inflammation.

## 2. Materials and Methods

### 2.1. Animals

6-week-old male ApoE-gene-knockout mice (strain C57/6J, introduced from The Jackson Laboratory, USA, and bred by the Laboratory Animal Center of Beijing University) weighing 19 to 21 g were fed from the age of 6 weeks onward with a high-fat diet containing 21% (wt/wt) fat and 1.25% (wt/wt) cholesterol that was sterilized by irradiation (^60^Coy). Mice were raised at clean level II, room temperature of 22 to 24°C, 50% relative humidity, and a 12-hour light/dark cycle. The study was approved by the Animal Research Committee of the China-Japan Friendship Hospital.

### 2.2. Medications

ShengjieTongyu granule was manufactured by the Chinese Herbal Medicine Pharmacy in China-Japan Friendship Hospital. Radix Astragalus, Rhizoma anemarrhenae, *Platycodon grandiflorum*, Cimicifuga foetida L, Bupleurum, Rhizoma sparganii, Curcuma zedoary, and so forth, were boiled together, and then dried to make granules. Each gram of granule was extracted from 4 grams of crude drug. Each bag contained 9 g granule. Simvastatin was provided by MSD Hangzhou, China. Normal saline wasobtained from the Beijing Double-crane Pharmaceutical Co., Ltd., (cat. 1009222).

### 2.3. Methods

#### 2.3.1. Grouping and Administration Methods

Atherosclerotic plaques were present in the mice at 16 weeks of age, and at this age animals were then randomized into 3 groups of 20 mice each: Shengjie Tongyu granule group, simvastatin group, and normal saline group (blank treatment group). The dosages of simvastatin and Shengjie Tongyu granule that were used were based on the clinical daily usual dosages for adults with a dose conversion coefficient of 9.01 and were the following: Shengjie Tongyu granule group: 4.00 g/kg/d, simvastatin group: 9.01 mg/kg/d, and normal saline group: 0.2 mL/d. Medications were dissolved in distilled water and administered once daily by gastric lavage. Ten mice in each group were killed for measurements at 30 weeks and 40 weeks of age, time periods chosen to illustrate the early and the later, more chronic, development of atherosclerosis and vascular remodeling.

#### 2.3.2. Evaluation of Aortic Vascular Remodeling

**Aorta Preparation**

Mice were killed after anesthesia with sodium barbital, and hearts and aortas were harvested under sterile conditions. In each group, ten aortas were fixed in 10% formalin for histological and immunohistochemical observation, and the other ten aortas were prepared as frozen slices and immediately stored in a −80°C freezer for Western blotting.

**Evaluation of Vascular Remodeling in the Aortic Root**

Aortic slices were collected consecutively from the beginning of the aortic valve to the point where the aortic valve disappeared (50 *μ*m length and 5 *μ*m slice thickness). Average plaque areas, lumen areas, and their ratios were measured in these slices after hematoxylin andeosin (HE) staining.

**Evaluation of Vascular Remodeling in the Aorta**

**Figure 1:**
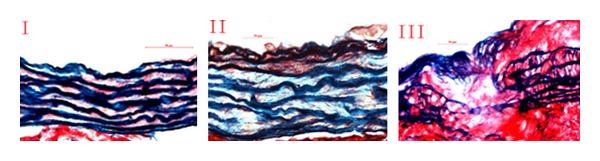
Images of different grades of aortic elastic fiber lesions. Figure 1(I), (II), and (III) represent grades I, II, and III lesions of elastic fibers. Grade I: elastic fibers are closely arranged, complete, and continuous (Figure 1(I)); grade II: the gaps between fibers are enlarged, and elastic fibers are partially ruptured (Figure 1(II)); grade III: elastic fibers are seriously damaged, fragmented, or absent (Figure 1(III)).

Vascular remodeling in the aortawas evaluated in a 3 mm section on the right side beneath the opening of the innominate artery. Slices were taken every 50 *μ*m, and 8 slices were prepared from each mouse. The slices were stained with Sirius red and Victoria blue, elastic fibers lesions of different grades as well as the content of type I and type III collagens were measured. Elastic fibers were graded according to the following classification standards. Grade I: elastic fibers are closely arranged, complete, and continuous (Figure 1(I)); grade II: the gaps between fibers are enlarged, and elastic fibers are partially ruptured (Figure 1(II)); grade III: elastic fibers are seriously damaged, fragmented, or absent (Figure 1(III)).

#### 2.3.3. Biochemical Determinations

**Determination of Blood Lipids and Serum Soluble ICAM-1 Level**

At 30 and 40 weeks of age, after the mice in each group were killed, blood from the inferior vena cava was collected, and the serum separated and stored at −80°C. Total cholesterol (TC), triglycerides (TG), and high-density lipoprotein cholesterol (HDL-C) were measured by enzymatic assay. Soluble ICAM-1 was measured using an ELISA reagent kit according to the manufacturer's instructions.

**Determination of Collagen Contents**

The collagen contents in the ascending aorta in ApoE-gene-knockout mice were measured by an immunohistochemical staining method.

**Monocyte/Macrophage Antibody-2 (MOMA-2) and Superoxide Anion Determination**

For the determination of MOMA-2, frozen slices of aorta were fixed in acetone and incubated with rat monoclonal anti-mouse MOMA-2 (Abcam, UK) at a dilution of 1 : 50 at room temperature. After washing, the slices were incubated in the dark with fluoresce in isothiocyanate (FITC)-labeled secondary antibody at room temperature. The results were observed under a fluorescence microscope (Nikon 90i, Japan). For determination of superoxide anions, the slices were incubated with superoxide anion probe (Abcam, UK, 2 *μ*mol/L) for 30 minutes, washed four times with PBS, and examined with a fluorescence microscope (Nikon 90i, Japan). Images were analyzed by an investigator who was blinded to the study protocol, using Image-ProP1us 5.1 software. Expression levels were quantified by calculating average optical density values.

**Western Blot Analysis of Aortic of NF*κ*B p50**

The aortic arch from each group was added to the protein lysis buffer and homogenized. Soluble proteins were recovered using centrifugation. The soluble proteins then underwent SDS polyacrylamide gel electrophoresis, and the separated proteins were transferred to polyvinylidene fluoride (PVDF) membranes. The membrane was blocked with 3% skim milk powder in washing buffer solution (10 mmol/L, Tris-HCl, and pH 7.6) for 1 hour at room temperature, followed by incubation overnight at 4°C with rabbit anti-mouse NF*κ*B P50 polyclonal antibody (1 : 600 dilution; Cell Signaling, USA), and goat anti-rabbit antibody (1 : 600) for 1.5 hours at room temperature successively. After each incubation, four 10-minute washes with PBS were performed. Thereafter, an Opti-4CN Substrate Kit, a chemiluminescence enhancer, was used to develop color in PVDF membranes. After incubation at room temperature for 20 minutes, a laser densitometer (Bio-Rad, USA) was used for gray-scale analysis of NF*κ*B P50 expression.

### 2.4. Statistical Analysis

Continuous variables are expressed as mean and standard deviation. Two-way ANOVA was performed to evaluate the effect of age and treatment and the interaction effect between age and treatment. Post hoc tests were performed by using Scheffe's method. The statistical significance level was set as 0.05. Statistical analyses were performed with SPSS 15.0 statistics software (SPSS Inc., Chicago, IL, USA).

## 3. Results

### 3.1. Mortality

No mice died in the period before 30 weeks of age; however, six mice died in the period between 30 and 40 weeks of age. No significant difference in mortality between groups (2 mice died in each group) was observed.

### 3.2. Progression of Atherosclerosis in Control Mice

In the control mice, as expected from the ApoE-gene-knockout mice genotype, plasma lipid concentrations increased with time and were significantly higher in 40-week-old than in 30-week-old mice. Vascular remodeling also increased, as shown by the increase in luminal area and percent of luminal area occupied by plaque, the deterioration of elastic fibers, and the shift from type I to type III collagen. Accompanying this increase in vascular remodeling was an increase in the inflammatory biomarkers, superoxide anion, ICAM-1, MOMA-2, and NF*κ*B.

### 3.3. Lipid Levels, Kidney, and Liver Function

Blood lipid levels increased with time in all groups and were significantly higher at 40 weeks than at 30 weeks. Neither of the two treatments had any significant effect on blood lipid levels at either time period (Table 1). Nor did either of the treatments have any significant effects on kidney of liver function (Table 2).

### 3.4. Cardiac Remodeling

#### 3.4.1. Lumenal and Plaque Areas

**Figure 2:**
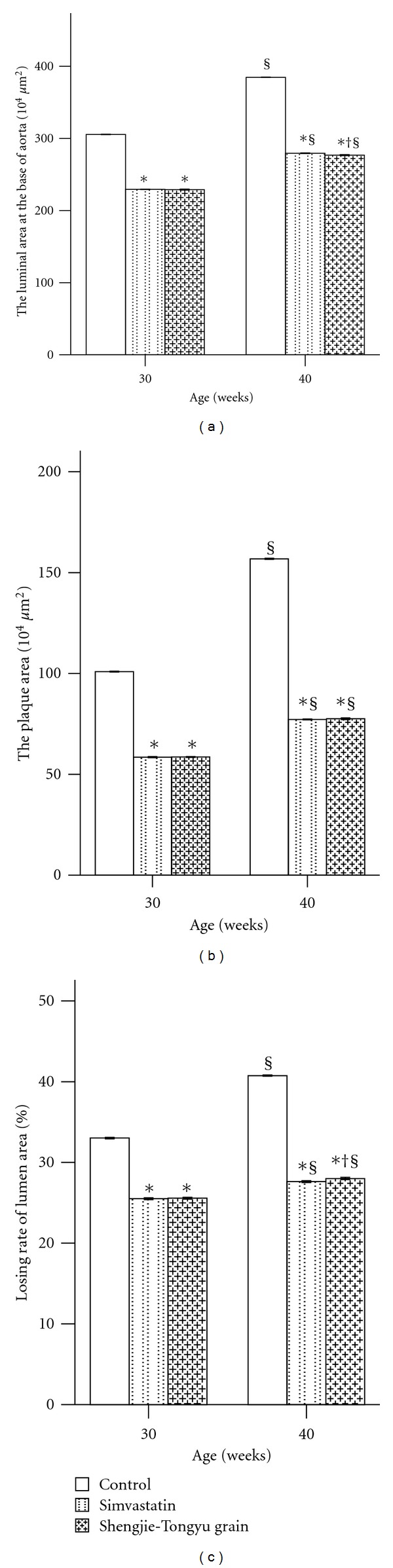
Effect of Shengjie Tongyu granule and simvastatin on aortic morphology in ApoE-gene-knockout mice. *Indicates a significant difference compared to the control group. ^§^Indicates a significant difference compared to the 30-week group. Lumenal area was measured using the outer elastic fiber as the boundary, and plaque area was measured from the outer layer of elastic fiber to the inside of the lumen. Loss rate as per cent of lumenal area was calculated as follows: loss rate = (plaque area / lumen area) × 100%.

**Figure 3:**
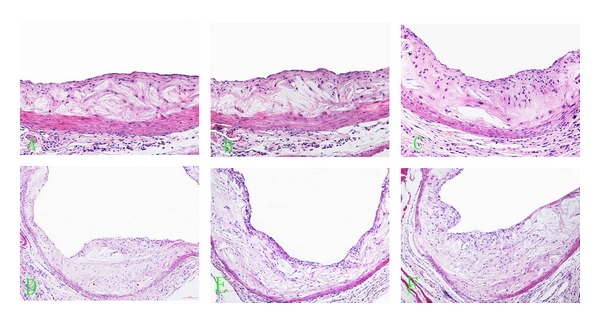
HE-stained images showing lumen at the base of aorta and plaque area during vascular remodeling in mice fed with Shengjie Tongyu, simvastatin or control, at 30 and 40 weeks. (A) Shengjie Tongyu group at 30 weeks; (B) simvastatin group at 30 weeks; (C) control group at 30 weeks; (D) Shengjie Tongyu group at 40 weeks; (E) simvastatin group at 40 weeks; (F) control group at 40 weeks. Magnification: ×200 (A, B, and C); ×100 (D, E, and F).

Lumenal area at the base of the aorta increased significantly with time in all groups, as did plaque area, both when measured alone or as a percentage of the luminal area (Figure 2). Mice treated with simvastatin or with Shengjie Tongyu granule had significantly smaller lumenal areas (30 weeks: 229.4 and 229.1 versus 305.6, *P* values < 0.001; 40 weeks: 279.5 and 276.9 versus 384.8, *P* values < 0.001) and plaque areas (30 weeks: 58.5 and 58.6 versus 100.9; 40 weeks: 77.2 and 77.5 versus 156.8, *P* values < 0.001) and a lower percentage of the lumen occupied by plaque (30 weeks: 25.5 and 25.6 versus 33.0, 40 weeks: 27.6 and 28.0 versus 40.8, *P* values < 0.001) than control mice.  Figure 3 shows the representative images of lumen and plaque in different groups at 30 and 40 weeks.

#### 3.4.2. Elastic Fibers and Collagen

**Figure 4:**
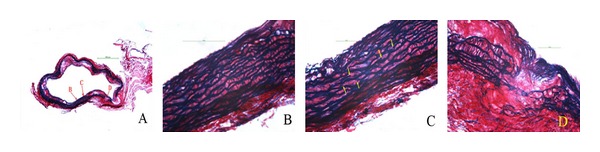
(A) Section of atherosclerotic aorta stained with Sirius red and Victoria blue. Enlargements of locations B, C, and D are shown on the right: (B) grade I lesion of elastic fibers; (C) grade II lesion with yellow arrows indicating the location of elastic fiber plaque rupture; (D) grade III lesion showing complete rupture of elastic plaque. Magnification of figure A (×40), B, C, and D (×400).

**Figure 5:**
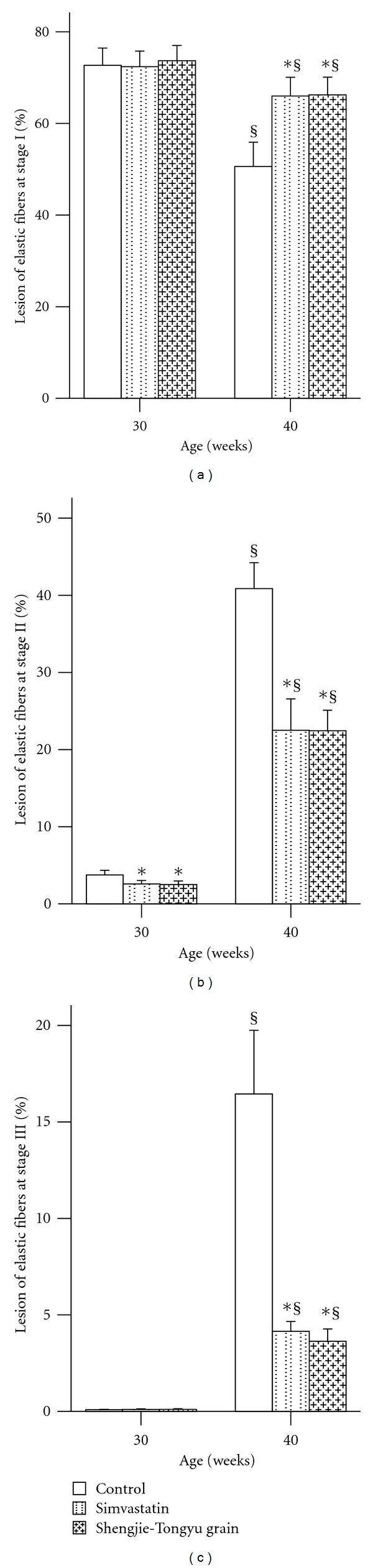
Effect of Shengjie Tongyu granule and simvastatin on elastic fiber of ascending aortic lesions at stage I (a), stage II (b), and stage III (c) in ApoE-gene-knockout mice. *Indicates a significant difference compared to the control group. ^§^Indicates a significant difference compared to the 30-week group.

Figure 4 shows a section of atherosclerotic aorta (A), illustrating type I (B), type II (C), and type III (D) lesions of elastic fibers. At 30 weeks, very little damage to elastic fibers was seen, either in control or drug-treated mice. (Figure 5). At 40 weeks, the percentage of undamaged (Type I) fibers decreased, and the percentage of moderately damaged (Type II) and strongly damaged (Type III) fibers increased; however, simvastatin and Shengjie Tongyu granule-treated mice had a smaller decrease in undamaged fibers (66.0 and 66.3 versus 50.6, *P *values < 0.001), and a smaller increase in damaged fibers (stage II: 22.5 and 22.4 versus 40.9, stage III: 4.2 and 3.6 versus 16.5, *P *values < 0.001) than control mice (Figure 5).

**Figure 6:**
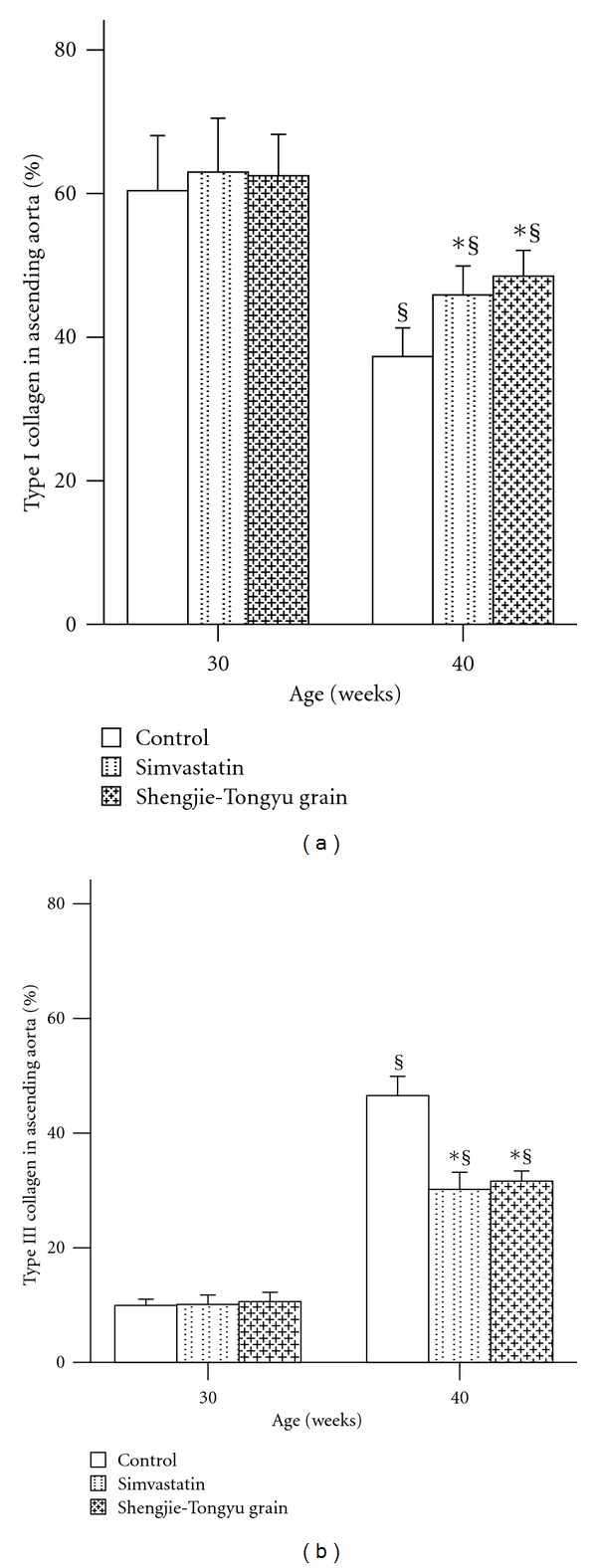
Effect of Shengjie Tongyu granule and simvastatin on collagen types in the ascending aorta in ApoE-gene-knockout mice. *Indicates a significant difference compared to the control group. ^§^Indicates a significant difference compared to the 30-week group. % indicates the percentage of type I collagen in all types collagen, or the percentage of type III collagen in all types of collagen.

**Figure 7:**
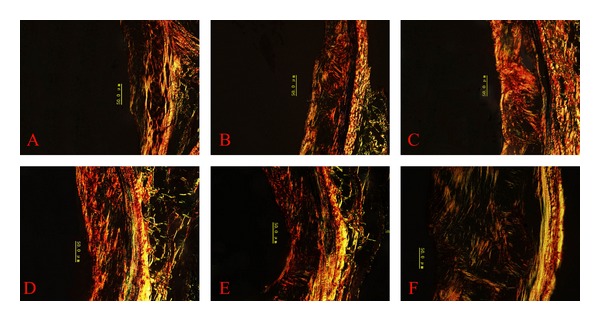
Aortic collagen I and III content. (A) Shengjie Tongyu group at 30 weeks; (B) simvastatin group at 30 weeks; (C) control group at 30 weeks; (D) Shengjie Tongyu group at 40 weeks; (E) simvastatin group at 40 weeks; (F) control group at 40 weeks. The photographs were taken using Sirius red staining with polarized light, under a magnification of ×400. Green color indicates type III collagen, and red-yellow indicates type I collagen.

Collagens showed a shift from type I to type III collagen at 40 weeks in all groups, but this shift was smaller in simvastatin and Shengjie Tongyu granule-treated animals (Figure 6).  Figure 7 shows representative illustrations of Shengjie Tongyu, simvastatin, and control collagen content at 30 weeks (A, B, and C) and 40 weeks (D, E, and F).

### 3.5. Markers of Inflammation

#### 3.5.1. ICAM-1

**Figure 8:**
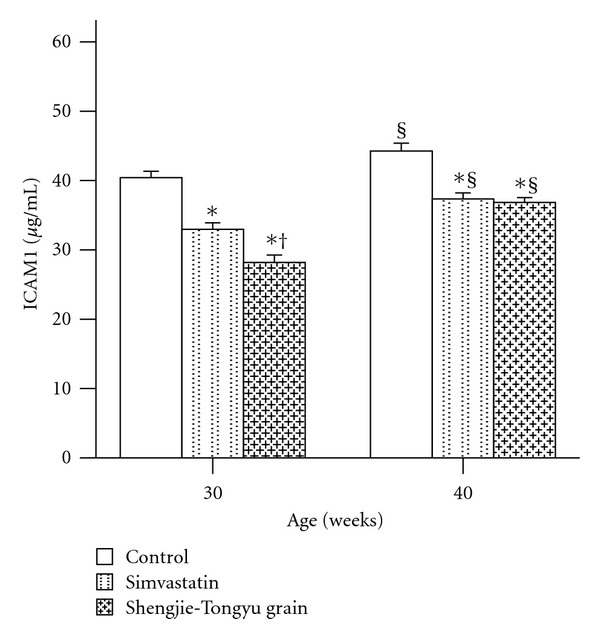
Effect of Shengjie Tongyu granule and simvastatin on plasma ICM-1 in ApoE-gene-knockout mice. *Indicates a significant difference compared to the control group. ^†^Indicates a significant difference compared to the simvastatin group. ^§^Indicates a significant difference compared to the 30-weeks group.

ICAM-1 levels increased with time in all groups and were significantly higher at 40 weeks than at 30 weeks. Simvastatin and Shengjie Tongyu granule treatment decreased ICAM-1 levels at both time periods (*P* < 0.001). At 30, but not at 40 weeks, Shengjie Tongyu granule treatment resulted in significantly lower levels of ICAM-1 than simvastatin treatment (Figure 8).

#### 3.5.2. Superoxide Anion

**Figure 9:**
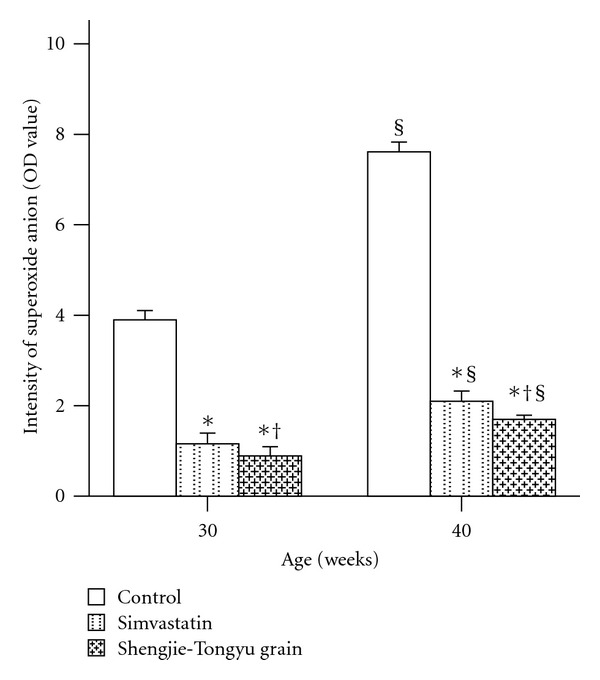
Effect of Shengjie Tongyu granule and simvastatin onsuperoxide anion content in ApoE-gene-knockout mice. *Indicates a significant difference compared to the control group. ^†^Indicates a significant difference compared to the simvastatin group. ^§^Indicates a significant difference compared to the 30-week group.

**Figure 10:**
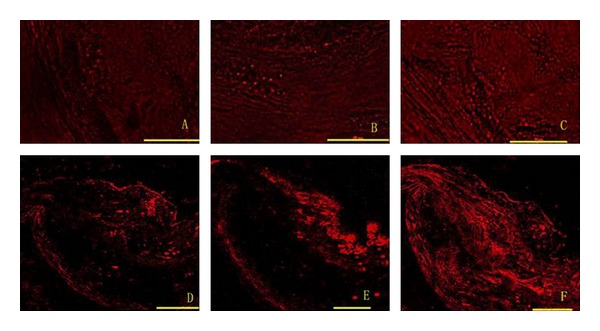
Expression of superoxide anion. (A) Shengjie Tongyu granule group at 30 weeks; (B) simvastatin group at 30 weeks; (C) control group at 30 weeks; (D) Shengjie Tongyu granule group at 40 weeks; (E) simvastatin at 40 weeks; (F) control group at 40 weeks. Scale bar: 50 um. Magnification: ×400.

Superoxide anion levels increased with time in all groups and were significantly lower in the simvastatin and Shengjie Tongyu groups than in the control group (30 weeks: 1.2 and 0.9 versus 3.9, 40 weeks: 2.1 and 1.7 versus 7.6, *P* values < 0.001). They were also lower in the Shengjie Tongyu group than in the simvastatin group (30 weeks: 0.9 versus 1.2, *P* value = 0.032; 40 weeks: 1.7 versus 2.1, *P* value = 0.002) (Figure 9).  Figure 10 shows illustrations of superoxide anion levels in Shengjie Tongyu granule, simvastatin, and control groups at 30 weeks (A, B, and C) and 40 weeks (D, E, and F).

**MOMA-2 Expression Level**

**Figure 11:**
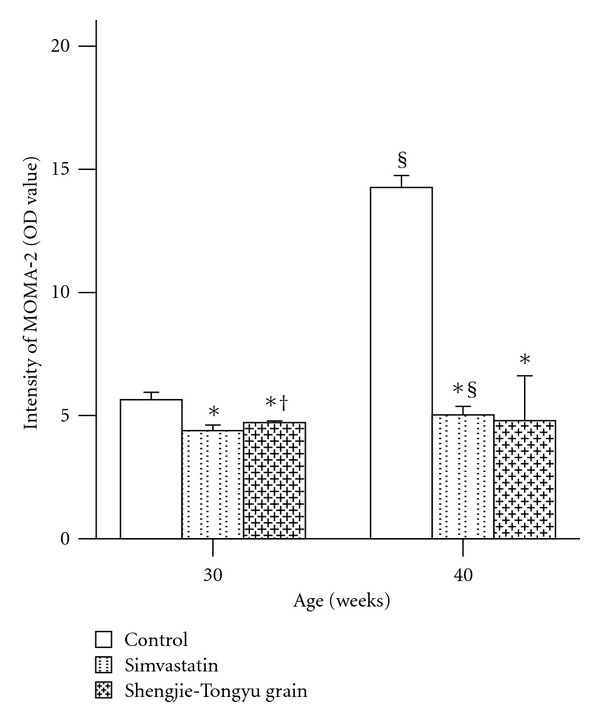
Effect of Shengjie Tongyu granule and simvastatin onMOMA-2 expression in ApoE-gene-knockout mice. *Indicates a significant difference compared to the control group. ^†^Indicates a significant difference compared to the simvastatin group. ^§^indicates a significant difference compared to the 30-week group.

**Figure 12:**
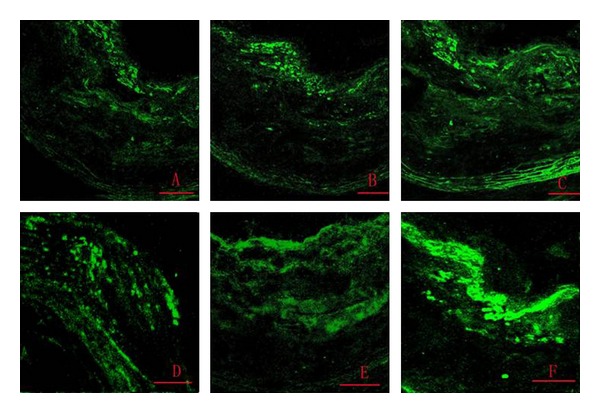
Expression of MOMA-2. (A) Shengjie Tongyu granule group at 30 weeks; (B) simvastatin group at 30 weeks; (C) control group at 30 weeks; (D) Shengjie Tongyu granule group at 40 weeks; (E) simvastatin at 40 weeks; (F) control group at 40 weeks. Scale bar: 50 um. Magnification: ×400.

MOMA-2 expression levels increased with time in all groups, but increased significantly less in the simvastatin and Shengjie Tongyu groups than in the control group (30 weeks: 4.4 and 4.7 versus 5.6, 40 weeks: 5.0 and 4.8 versus 14.3, *P* values < 0.001). At 30 weeks, MOMA-2 expression in the Shengjie Tongyu group was higher than in the simvastatin group (4.7 versus 4.4, *P* value = 0.012) (Figure 11).  Figure 12 shows illustrations of these changes.

**NF*κ*B P50 Expression**

**Figure 13:**
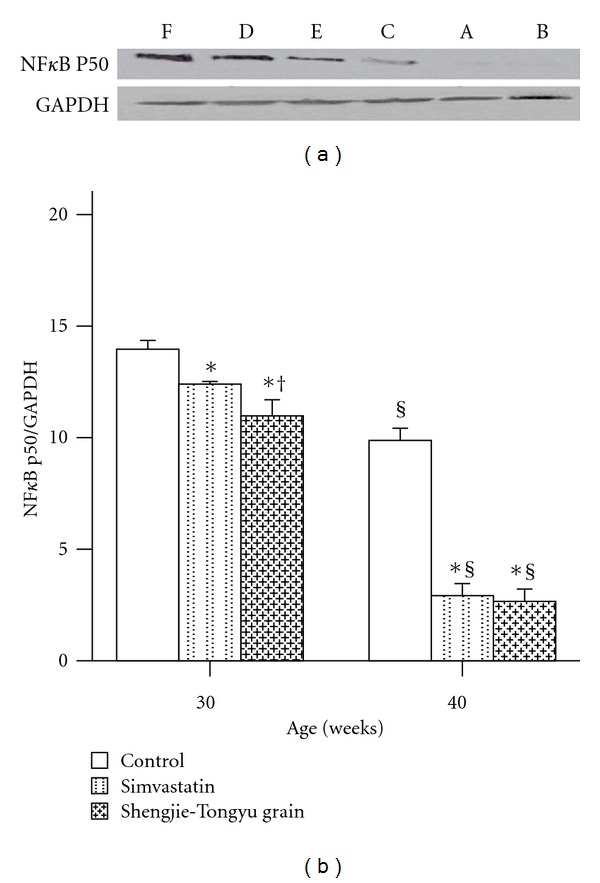
Effect of Shengjie Tongyu granule and simvastatin on the expression of NF*κ*B p50 in the aorta in ApoE-gene-knockout mice. Lanes A, B, and C indicate the protein expression of NF*κ*B p50 in 30-week-old mice in control; simvastatin and Shengjie Tongyu granule groups, respectively. Lanes D, E, and F indicate the protein expression of NF*κ*B p50 in 40-week-old mice in control; simvastatin and Shengjie Tongyu granule groups, respectively. *Indicates a significant difference compared to the control group. ^†^Indicates a significant difference compared to the simvastatin group. ^§^Indicates a significant difference compared to the 30-week group.

The NF*κ*B P50 expression level increased with time in all groups, but was significantly lower in the simvastatin and Shengjie Tongyu groups than in the control group (30 weeks: 12.4 and 11.0 versus 14.0, 40 weeks: 2.9 and 2.7 versus 9.9, *P* values < 0.001). At 30 weeks, NF*κ*B P50 expression in the Shengjie Tongyu group was significantly lower than in the simvastatin group (11.0 versus 12.4, *P *value < 0.001) (Figure 13).

## 4. Discussion

Shengjie Tongyu granule and simvastatin decreased both vascular remodeling and signs of vascular inflammationin ApoE-gene-knockout mice fed a high fat diet. However, neither treatment decreased the elevated blood lipid concentrations seen in this model.

The four markers of inflammation monitored each acted at a different stage in the inflammatory process: (1) influx of inflammatory cells (MOMA-2), (2) endothelial binding and transmigration of inflammatory cells (ICAM-1), (3) increase in reactive oxygen species (superoxide anion), and (4) increase in a mediator of an intracellular pathway activated in inflammation (NF*κ*B). At 30 weeks, superoxide anion was decreased to about 25% of control levels by both treatments, while the other three markers remained at 75 to 85% of control levels. At 40 weeks, a more chronic stage of atherosclerosis, ICAM-1 levels remained at about 80% of control, but MOMA-2 and NF*κ*B levels in treated animals dropped to 34% and 28% of control, that is, inhibition of these markers became similar to the inhibition of superoxide anion.

Atherosclerosis is a chronic inflammatory process, and the inflammatory cells involved produce a large amount of ROS during this process. ROS impairs cell functions, leading to the activation and expression of NF-*κ*B. Studies have shown that ROS production and increased NF-*κ*B expression occur during the early stage of atherosclerosis [17]. Then, as the development of atherosclerosis progresses and inflammation continues, ox-LDL produced from oxidized ROS may cause additional NF-*κ*B activation. In our study, the reason that it took longer for the two treatments to decrease NF-*κ*B levels than to decrease ROS might be because of the secondary ox-LDL effect.

Superoxide anions can activate downstream MAPKs, Janus kinase, STAT, and NF-*κ* [18–20]. The current results suggest that simvastatin and Shengjie Tongyu granule act by decreasing superoxide anion and in this way decrease NF*κ*B. But the pathology leading to atherosclerosis in complex, and other pathways and mechanisms cannot be ruled out. Recently simvastatin was shown to decrease vascular remodeling by inhibiting the volume-operated chloride channel [21], but this study was of vascular remodeling induced by hypertension, and may not be applicable to the model used here, that is, to vascular remodeling as a result of atherosclerosis. Our results on the actions of simvastatin in atherosclerosis are, however, consistent with those of others [5]. 

Shengjie Tongyu granule depressed ICAM-1, superoxide anion, and NF*κ*B slightly more and MOMA-2 slightly less than simvastatin. But in general, the actions of simvastatin and Shengjie Tongyu granule on each of the parameters studied were very similar. This result is somewhat surprising because simvastatin is a single defined chemical, and Shengjie Tongyu is composed of a number of components with different actions. This result could be explained logically if the two substances had identical effects on a single key instigating factor (such as superoxide anion), and the other phenomena observed were merely sequels to the initial action. Of course, only further study can determine whether this insight is true.

The present study offers a new understanding of the mechanism and targets for Shengjie Tongyu granule action in atherosclerosis that may be helpful in further development of methods for the prevention and treatment of vascular remodeling in atherosclerosis. In the future, we hope to investigate which specific compound in Shengjie Tongyu granule has the inhibitory effect on inflammation and vascular remodeling described here.

## Figures and Tables

**Table 1:** Blood lipids.

		Treatment group			
		Control	Shengjie Tongyu granule	Simvastatin	*P* value^a^
Cholesterol (mmol/L)					
Age group	30 weeks	16.0 ± 3.6	16.3 ± 3.3	14.9 ± 2.4	0.452
	40 weeks	13.6 ± 4.1	15.4 ± 3.6	16.4 ± 3.4	
	*P* value^b^		0.382		

TG (mmol/L)					
Age group	30 weeks	1.92 ± 0.42	1.92 ± 0.22	2.10 ± 0.34	0.336
	40 weeks	2.19 ± 0.32	2.07 ± 0.31	2.11 ± 0.33	
	*P* value^b^		0.029*		

HDL (mmol/L)					
Age group	30 weeks	3.2 ± 0.3	3.2 ± 0.2	3.2 ± 0.3	0.436
	40 weeks	3.2 ± 0.2	3.1 ± 0.3	3.1 ± 0.3	
	*P* value^ b^		0.141		

LDL (mmol/L)					
Age group	30 weeks	12.5 ± 3.5	12.9 ± 3.6	11.4 ± 2.3	0.416
	40 weeks	10.0 ± 3.9	11.9 ± 3.4	12.9 ± 3.3	
	*P* value^b^		0.329		

TG: triglycerides; HDL: high-density lipoprotein; LDL: low-density lipoprotein. ^a^For the effect of treatment group when age was fixed; ^b^for the effect of age when treatment group was fixed. *P < 0.05 indicates a significant age effect was observed.

**Table 2:** Effects of Shengjie Tongyu granule and simvastatin on kidney and liver function.

		Treatment group			
		Control	Shengjie Tongyu granule	Simvastatin	*P *value
Alt (IU/L)					
Age group	30 weeks	18.20 ± 2.75	18.00 ± 3.37	23.70 ± 6.55	> 0.05
	40 weeks	26.30 ± 6.85	26.10 ± 7.59	16.40 ± 3.4	

T-Bil (*μ*mol/L)					
Age group	30 weeks	9.76 ± 3.50	10.35 ± 3.82	9.99 ± 3.63	> 0.05
	40 weeks	11.69 ± 3.30	12.25 ± 3.76	11.94 ± 3.67	

D-Bil (*μ*mol/L)					
Age group	30 weeks	3.55 ± 1.47	3.82 ± 1.61	3.78 ± 1.37	> 0.05
	40 weeks	4.32 ± 1.56	4.50 ± 1.31	4.31 ± 1.59	

Cr (*μ*mol/L)					
Age group	30 weeks	5.35 ± 1.23	5.31 ± 1.01	5.40 ± 1.26	> 0.05
	40 weeks	5.91 ± 1.40	5.23 ± 1.47	5.72 ± 1.29	

BUN (mmol/L)					
Age group	30 weeks	81.60 ± 10.57	80.91 ± 8.48	81.92 ± 8.86	> 0.05
	40 weeks	85.82 ± 9.17	79.99 ± 9.56	83.51 ± 7.81	

Alt: alanine aminotransferase; T-Bil: total bilirubin; D-Bil: direct bilirubin. BUN: blood urea nitrogen; Cr: creatinine. *P* > 0.05 indicates no significant difference was observed.
